# Evolutionary computation: the next major transition of artificial intelligence?

**DOI:** 10.1186/s13040-017-0147-3

**Published:** 2017-07-29

**Authors:** Moshe Sipper, Randal S. Olson, Jason H. Moore

**Affiliations:** 1 Institute for Biomedical Informatics, University of Pennsylvania, Philadelphia, 19104–6021 PA USA; 2 Department of Computer Science, Ben-Gurion University, Beer Sheva, 8410501 Israel

## Editorial

Artificial intelligence (AI), a broad field that deals with the ongoing pursuit to render machines capable of performing intelligent tasks, has taken the academic and industrial worlds by storm in a breathtakingly short time span. These days, when you engage in the daily surf of your favorite news website, some mention of AI will probably ensue. Machine learning, currently the most prominent subfield of AI, focuses on algorithms that learn from data, with deep learning—employing artificial neural networks with several hidden layers—being the jewel in the crown.

From playing Go to processing radiological images, machine learning’s success and breadth of scope is undeniable. Yet we mustn’t forget that the parent field of AI has birthed many other offspring. In particular, we wish to shine a light on the field of *evolutionary computation (EC)*, which we believe is poised to be “The Next Big Thing”.

In EC, core concepts from evolutionary biology—inheritance, random variation, and selection—are harnessed in algorithms that are applied to complex computational problems. The field of EC, whose origins can be traced back to the 1950s and 60s, has come into its own over the past decade. EC techniques have been shown to solve numerous difficult problems from widely diverse domains, in particular producing human-competitive machine intelligence [1]. As argued by the authors of this latter paper, “Surpassing humans in the ability to solve complex problems is a *grand challenge*, with potentially far-reaching, transformative implications.”

EC is applicable over a wide range of problem categories, including classification, regression, clustering, design, optimization, planning, and generating computer programs. Moreover, the range of applications for which EC has worked well is staggering, including such disparate domains as antenna design [2], generating winning game strategies [3], automated program improvement [4], and bioinformatics [5].

EC presents many important benefits over popular deep learning methods:

- EC relies to a far lesser extent on the existence of a known or discoverable gradient within the search space.
- EC thrives when applied to design problems, where the objective is to design new entities from scratch (e.g., antennas [2] and game strategies [3]).
- EC algorithms require fewer a priori assumptions regarding the problem being investigated.
- However, EC seamlessly lends itself to the integration of human expert knowledge as needed (e.g, [3]).
- EC can solve problems with no known solutions, where human expertise is limited or absent altogether [6].
- EC has proven to work well in combination with many other AI techniques, including artificial neural networks [7] and other machine learning algorithms [8].
- EC algorithms are inherently distributed, and are ripe for running in parallel on multi-core or distributed cloud-computing systems [9].
- EC algorithms are anytime algorithms, meaning that they can provide a reasonable solution to a problem even when prematurely interrupted.
- The representation of solutions in EC algorithms can be quite flexible, which lends itself to highly interpretable models if interpretable solution representations are used.
- EC algorithms require little to no data to solve a problem; they can provide a solution based on any criteria in the fitness function.
- Several EC algorithms can beautifully handle multiple objectives [10].
- EC is conceptually simple and easy for non-experts to learn and apply.

Fogel [11] discusses additional benefits of EC, while [12] cogently presents EC’s advantages from an industrial perspective.

For these reasons we believe that EC is poised to rise to prominence in the near future, with evolutionary algorithms put to use far more than they are today. This development will come as no surprise to seasoned EC practitioners, who have been aware of the merits of evolution for a very long time. After all, since evolution by natural selection has given rise to human intelligence, surely artificial intelligence will greatly benefit from this process.

## Abbreviations

AI: Artificial intelligence
EC: Evolutionary computation
